# Secular and Religious Social Support Better Protect Blacks than Whites against Depressive Symptoms

**DOI:** 10.3390/bs8050046

**Published:** 2018-05-04

**Authors:** Shervin Assari, Maryam Moghani Lankarani

**Affiliations:** 1 Department of Psychiatry, University of Michigan, Ann Arbor, MI 48109, USA; 2 Center for Research on Ethnicity, Culture and Health, School of Public Health, University of Michigan, Ann Arbor, MI 48109, USA; 3 Medicine and Health Promotion Institute, Tehran, Iran; lankaranii@yahoo.com

**Keywords:** population groups, ethnic groups, African Americans, social support, religion, depressive symptoms, depression

## Abstract

**Purpose:** Although the protective effect of social support against depression is well known, limited information exists on racial differences in this association. The current study examined Black-White differences in the effects of religious and secular emotional social support on depressive symptoms in a national sample of older adults in the United States. **Methods:** With a longitudinal prospective design, the Religion, Aging and Health Survey, 2001–2004, followed 1493 Black (*n* = 734) and White (*n* = 759) elderly individuals (age 66 and older) for three years. Race, demographics (age and gender), socio-economics (education and marital status) and frequency of church attendance were measured at baseline in 2001. Secular social support, religious social support, chronic medical conditions and depressive symptoms [8- item Center for Epidemiological Studies-Depression scale (CES-D)] were measured in 2004. Multiple linear regression models were used for data analysis. Results: In the pooled sample, secular and religious social support were both protective against depressive symptoms, net of all covariates. Race interacted with secular (*β* = −0.62 for interaction) and religious (*β* = −0.21 for interaction) social support on baseline depressive symptoms (*p* < 0.05 for both interactions), suggesting larger protections for Blacks compared to Whites. In race-specific models, the regression weight for the effect of secular social support on depressive symptoms was larger for Blacks (*β* = −0.64) than Whites (*β* = −0.16). **Conclusion:** We found Black—White differences in the protective effects of secular and religious social support against depressive symptoms. Blacks seem to benefit more from the same level of emotional social support, regardless of its source, compared to Whites.

## 1. Introduction

Paradoxically, despite being more frequently exposed to a wide range of stressors, Blacks have a lower risk of depression compared to Whites [1,2,3,4,5,6,7,8,9,10,11,12,13]. Also known as the *Black-White mental health paradox*, this phenomenon is one of the mysteries of racial and ethnic mental health research in the United States [4,5,6,7,8,14]. Many researchers have tried to find possible explanations for this paradox. Keyes has suggested that Blacks may experience psychological growth and flourishing due to social and economic adversities [4].

Built on differential effects hypothesis [15,16], we have shown that Blacks and Whites differently gain health from the economic resources that become available to them [16,17]. In this view, Blacks gain less from economic resources that have not been available to them for decades [18,19,20,21,22]. In contrast, Blacks may have turned to non-economic resources that they could freely mobilize, to compensate lack of economic resources [23]. Among freely available resources to Blacks, even from the time of slavery, has been religion and religious social support [24]. In this view, not only Blacks are more religious than Whites, they have better learned to mobilize it to cope with stress and adversity [25,26].

In their landmark review paper in 1998, Ellison and Levin proposed several mechanisms to explain the effect of religion on health [27]. Central to their explanation was social support that could be used to buffer stress. Several other studies. This is also in line with other theoretical [28] and empirical [29,30,31] work on the protective effects of religious social support on health. Neal Krause has documented extensive evidence showing that social support is a main mechanism that explains the protective effects of religion on health [32,33,34]. Taylor and Chatters have also documented supportive relations in the church setting in the lives of Blacks [35,36,37,38,39]. Cohen’s social support theory also emphasizes [40], Protective effects of social support. These effects, however, vary across racial groups [25,26,41,42].

One potential explanation for this paradox is a higher availability and a higher efficacy of positive psychosocial and cognitive resources such as social support, family relations and religion in the life of Blacks compared to Whites [4,5,6,7,8]. For instance, Mouzen has used data from the National Survey of American Life (*n* = 4086) to examine whether family and religion explain the better mental health of Blacks, despite higher levels of adversities [7,8]. She, however, has found few race differences in the quantity and quality of friendships, fictive kinships and family relationships and such differences did not explain the race paradox in mental health [7,8].

In a series of attempts to understand the Black-White mental health paradox, we have documented major racial differences in the psychosocial and medical correlates of depression and depressive symptoms [5,43,44,45,46,47,48,49]. For instance, in two studies, depressive symptoms predicted mortality of Whites but not Blacks [50,51]. In other studies, depressive symptoms and negative affect predicted an increase in chronic medical conditions of Whites but not Blacks [6,52,53]. Baseline level of depressive symptoms was also found to predict subsequent risk of major depressive symptoms (MDD) among Whites but not Blacks [54]. Weaker effects of psychosocial risk factors on physical health outcomes in Blacks compared to Whites were not limited to depressive symptoms and were replicated for education, perceived control over life and self-rated health [6,18,40,50,52,53,54,55,56,57]. These patterns are indicative of a systemic resilience among Blacks [4,5].

One potential hypothesis that may explain the weaker effects of risk factors on Blacks is a higher availability of social support and religion [7,8,25,41]. In this view, social support and religiosity are not only more commonly found among Blacks, they are also more effective among them [41,42], making Blacks more resilient to the harmful effects of social and economic adversities compared to Whites. 

In 2013, we analyzed data from National Survey of American Life (NSAL) and showed that religious social support fully mediated the association between church attendance and life satisfaction for Blacks but not Whites. Authors concluded that religious social support may have more health benefits for Blacks compared to Whites [25]. On a nationwide sample of older adults, Krause documented stress-buffering effects of church-based support for Blacks but not Whites [42]. Lincoln, Chatters and Taylor conducted a secondary analysis of the National Comorbidity Survey (NCS) and found stronger protective effects of social support against psychological distress in Blacks compared to Whites [41]. All these studies are indicative of *Blacks’ faith advantage in health* [26].

We conducted this study to compare Black and White older adults for the effects of secular and religious emotional social support on depressive symptoms in the United States. In line with previous studies [25,41,42], we hypothesized stronger protective effects of religious and secular social support against depressive symptoms in Black than White older Americans. 

## 2. Methods

### 2.1. Design, Setting and Sampling

The Religion, Aging and Health Survey, 2001–2004, is a national household longitudinal study of Black and White older adults in the United States [58,59]. The study participants were 1500 older adults who were either White or Black older adults. The study only sampled non-institutionalized English speaking Americans with more than 65 years of age. Participants were either Christians or individuals who were not associated with any faith. The study oversampled older Blacks [58,59]. As a result, older Blacks represented half of the sample. The overall response rate for the baseline interviews was 62%. Geographically, the study population was restricted to all eligible persons residing in the coterminous United States (i.e., residents of Alaska and Hawaii were excluded). The sampling frame of this study consisted of all eligible persons contained in the Health Care Finance Administration (HCFA) Medicare Beneficiary Eligibility List, currently called the Center for Medicare and Medicaid Services (CMMS). This list contains the name, gender and race of older individuals in the United States [58,59].

### 2.2. Measures

Age, gender, race, education and religious involvement were measured at baseline in 2001. Secular emotional social support, religious emotional social support, number of chronic medical conditions (12 chronic medical conditions), self-rated health (SRH) and depressive symptoms were measured in 2004. 

*Depressive symptoms.* Depressive symptoms were measured using the 8–item Center for Epidemiological Studies-Depression scale (CES-D) scale [60], which were administered both in 2001 and 2004. The measure included items to capture two domains of depression: negative emotions and somatic symptoms. This abbreviated CES-D measure has shown acceptable reliability and validity [61,62,63]. Some examples of the items included (1) I felt depressed; (2) I felt sad; and (3) I could not get going. Item responses were on a four Likert scale, ranging from 1 (“Rarely or none”) to 4 (“most or all of the time”). A mean score was calculated. Depressive score, with a potential range from 1 to 4, was treated as a continuous measure. Higher scores were indicative of more severe depressive symptoms [64,65].

*Frequency of Church Attendance.* Frequency of church attendance was measured in 2001 (Wave 1) using the following item: How often do you attend religious services? Responses were never (1); less than once a year (2); about once or twice a year (3); several times a year (4); about once a month (5); 2–3 times a month (6); nearly every week (7); every week (8); and several times a week (9) [66].

*Religious Social Support.* We measured religious emotional social support at Wave 2 with a 3–item measure developed by Krause (2002) [66,67]. The items included: (1) “Not counting your minister, pastor, or priest, how often does someone in your congregation let you know they love and care for you?”; (2) “How often does someone in your congregation talk with you about your private problems and concerns?”; and (3) “How often does someone in your congregation express interest and concern in your well-being?” These items were scored as follows: (1) never; (2) once in a while; (3) fairly often; and (4) very often. Authors calculated a mean score, ranging from 1 to 4, where a higher score on the scale indicates receiving more spiritual support from fellow church members. The reliability is high with a Cronbach’s alpha of 0.825 [68].

*Secular Social Support.* We used a 3–item measure developed by Krause (2002) to evaluate participants’ received emotional support from people outside of their congregations [67]. Items included: (1) “Not counting your minister or fellow church members, how often do your family and friends let you know they love and care for you”?; (2) “Not counting your minister or fellow church members, how often do your family and friends talk with you about your private problems and concerns?”; and (3) “Not counting your minister or fellow church members, how often do your family and friends express interest and concern in your well-being?.” These item responses were as follow: (1) never; (2) once in a while; (3) fairly often; and (4) very often. We computed the mean score as the total score, ranging from 1 to 4, with a higher score denoting more frequent secular emotional support. The measure had acceptable reliability with a Cronbach alpha estimate of 0.786 [68].

*Chronic Medical Conditions.* The presence of the following chronic medical conditions during the past 12 months was measured during Wave 2 in 2004: (1) hypertension; (2) heart problems; (3) diabetes; (4) cancer; (5) renal disease; (6) arthritis; (7) gastrointestinal disease; (8) chronic liver disease; (9) urinary tract disorders; (10) ophthalmological diseases; (11) respiratory conditions; and (12) other chronic health problems. Number of conditions potentially ranged between 0 and 12, with a higher score reflecting higher number of conditions [14].

### 2.3. Statistical Analysis

SPSS 22.0 for Windows (IBM Inc. Armonk, NY, USA) was used for data analysis. Multiple linear regression models were estimated in the pooled sample as well as specific to race. In all models, frequency of depressive symptoms was the dependent variable. We ran separate models with church attendance, religious social support and secular social support as independent variables. *Model 1* was conducted in the pooled sample and only included the main effects, without the interaction term. *Model 2* was tested in the pooled sample but also included the interaction term between race and the predictor of interest. *Model 3* and *Model 4* were specific to Whites (*Model 3*) and Blacks (*Model 4*). In all models, demographics, socio-economics and physical health were covariates. Race was the focal moderator. Standardized Beta with *p* values were reported. We considered *p* less than 0.05 as significant. Less than 5% of the data had a missing value.

### 2.4. Ethics

All participants provided written informed consent. All data were collected anonymously. The University of Michigan Institutional Review Board (IRB) approved the study protocol.

## 3. Results

The study followed 1493 older adults who were either Black (*n* = 734) or White (*n* = 759) (age 65 or older) for three years. Table 1 summarizes the descriptive statistics in the overall sample and also by race. Average age was similar across racial groups, whereas Blacks were more often female, had lower education and were less frequently married. Blacks more frequently attended church and reported higher secular and religious emotional social support, yet reported feeling more depressed (Table 1).

Table 2 shows a summary of four linear regression models in the pooled sample with (*Model 1*) and without (*Model 2*) interactions terms and also in Whites (*Model 3*) and Blacks (*Model 4*). High church attendance showed a protective effect against depressive symptoms in the pooled sample (*Model 1*) and race did not interact with the church attendance on depressive symptoms (*Model 2*). *Model 3* and *Model 4* also shows that church attendance protects both Whites and Blacks against depressive symptoms (Table 2).

Table 3 summarizes four linear regressions that show higher levels of religious social support is associated with lower levels of depressive symptoms, net of all covariates in the pooled sample (*Model 1*). A significant interaction was found between race and religious social support, suggesting larger effects for Blacks compared to Whites (*Model 2*). These interactions were significant, net of all covariates. *Model 3* and *Model 4* also showed that religious social support protects Whites and Blacks against depressive symptoms (Table 3).

**Figure 1:**
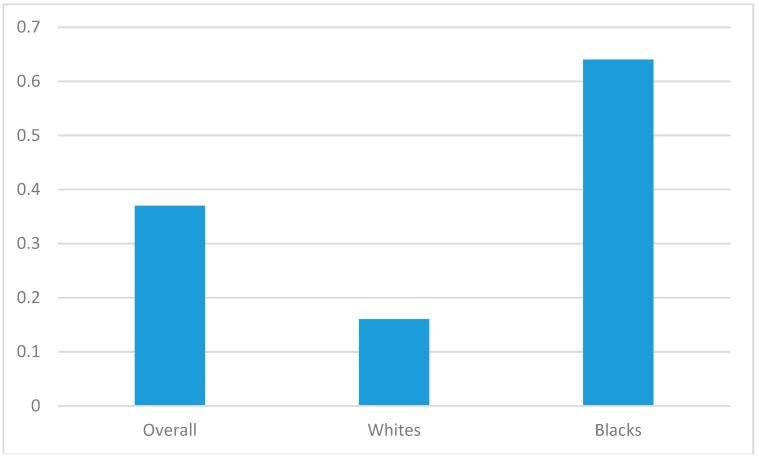
Protective effect of secular social support overall and by race. (Numbers are standardized regression coefficients for the effects of social support on depressive symptoms).

Table 4 provides a summary of the effects of secular social support on depressive symptoms among Blacks and Whites net of covariates. Higher levels of secular social support were associated with lower levels of depressive symptoms in the pooled sample (*Model 1*) and we found an interaction between race and secular social support, suggesting larger effects for Blacks compared to Whites (*Model 2*). Although significant for Whites (*Model 3*) and Blacks (*Model 4*), the regression coefficient for the effect of secular social support on depressive symptoms was larger for Blacks (*B* = −0.64) compared to Whites (*B* = −0.16) (Table 4, Figure 1).

## 4. Discussion

Our first finding suggests that church attendance, secular social support, as well as religious social support, were all protective against depressive symptoms among older adults. Our second finding showed Black—White differences in the effects of secular and religious social support on depressive symptoms, with Blacks benefiting more from the same level of emotional social support, regardless of its source, compared to Whites. Black and White older adults, however, did not differ in the protective effect of church attendance on depressive symptoms.

Our first finding in the pooled sample is in line with previous theoretical and empirical work. There are at least three theoretical perspectives on the health effects of social support: (1) the stress and coping perspective; (2) the social constructionist perspective; and (3) the relationship perspective. These models suggest that either directly, or through buffering the effects of stressors, via enhancing coping abilities, social support protects people against poor mental health including depression. Social support promotes self-esteem and self-regulation and coping and reduces perceived stress [69].

Our second result regarding the interaction between race and support on depressive symptoms is in harmony with previous findings by our team [25], Lincoln et al., [41] and Krause [70] on racial differences in mental health benefits associated with social support in the church setting. All of these studies have suggested that Blacks gain more health benefits from religion, compared to Whites [70]. Skarupski has called this phenomenon *Blacks’ faith advantage in health* [26]. Blacks and Whites have different transactions in their social networks inside and outside church settings [71,72,73,74] which may be in part due to their different network composition [74,75]. Our findings may be due to racial and ethnic differences in the organization and programmatic emphases of religious services and churches that have major implications for the religion–health link across racial groups [25,41]. Patterns and contents of religious activities differ for Blacks and Whites [76]. The structure and mission of church also differs for Blacks and Whites [77]. Such differences may result in stronger associations between emotional support in the context of religion and health outcomes [2,78]. Our finding is, however, does not support a stronger effect of social network on health of Whites compared to Blacks [79]. 

Self-construals may also help us understand our findings. Blacks and other minority groups have higher value orientations that are supportive of familyism, collectivism and interdependence. Higher interconnected cultural beliefs may result in a higher susceptibility of Blacks to the effects of social support [80]. Self-construals shape individuals’ approaches to their environment, including social ties [81]. In fact, interdependent or communal cultural systems support a sense of harmony control via establishing social networks of loyalty and support [82,83]. Blacks, who may have higher collectivistic attitudes, may place a higher priority on fostering social harmony; they may prefer to maintain relationships with others rather than control the situation, which may result in higher wellbeing [81]. These traits also shape how the individual maintains harmony, sense of control and coping skills; how the individual interacts with the contextual, social, or spiritual forces; and how the individual attempts to merge with or distinct from these forces [82,84]. Blacks who are from more communal or interdependent cultures, often value interpersonal relationships and have a higher tendency to see others as a part of the self. Whites are more often from cultures that endorse more independent attitudes and value uniqueness and distinction of self from others [81].

We found differential effects of social support for Blacks and Whites. As Lincoln et al. [41] argued in 2003, social factors operate differently across racial groups. They also mentioned that particular psychosocial constructs may have differential salience across race and ethnic groups. In addition, social and psychological processes may operate in unique ways for some racial and ethnic groups [41]. There is a need to study how racial groups differ in psychosocial processes that shape health and illness [41,85,86,87]. Failure to account for the unique population specific cultural factors that accompany the life circumstances of diverse racial group may result in biased findings. Researchers should not always assume that the same social theories and models similarly explain outcomes across diverse racial or ethnic groups [41]. Each racial or ethnic group may benefit or be vulnerable to specific set of risk and protective factors [41]. This is the argument behind the *differential effects hypothesis* [15]. Understanding nuances of the association between social support and depression is important for mental health promotion of populations. Low social support typically leads to the development or worsening of depression [88]. The findings of this study are in line with the literature on the protective effects of positive social relationships on physical and mental health [89,90,91,92]. The support individuals receive from others [91], the quality and quantity of their social interactions [93] and their feelings of isolation and loneliness [94] have major implications for their health and wellbeing [69,95].

Higher tendency for Blacks to receive social support from fellow church members is possibly due to scarcity of economic resources in their lives [16,17]. In response, Black communities have been historically seeking resilience in Black church [96]. As argued by Nelsen and Nelsen, in 1975, as a result of centuries of racism, slavery, prejudice and discrimination, church has become the center of the Black community [97]. Blacks have turned to the church primarily because for decades as Black church was almost the only institution that was built, funded and wholly owned by them. For Blacks, church has become more than a place of worship; it has also become a social institution for interchange of social services. Given the central role of church for Blacks, supportive social relationships with the fellow church members should be expected to have a stronger effect for Blacks than Whites 705. It has been suggested that church, as a social and religious body, serves as an ‘extended family’ for Blacks. Many Blacks who face adversities in daily life exchange support with their kin in church [98].

Stronger effects of secular and religious emotional social support on depressive symptoms in Blacks compared to Whites may explain the weaker health effects of psychical risk factors such as depressive symptoms, self-rated health and hostility on mortality risk for Blacks compared to Whites [50,55,56,57,73,74,99]. These studies have suggested that regardless of the type of predictor, psychological variables better predict physical health outcomes for Whites than Blacks [55,57,100]. The authors suggest that psychosocial resources may have stronger effects on depressive symptoms for Blacks compared to Whites. 

Major race differences exist in the complex associations between psychosocial resources such as socioeconomic status (SES), social support, stress, depression and physical health [43,44,51,56,101,102,103,104,105,106,107]. For instance, race and ethnicity modify the effects of SES resources on mortality [18,53,101,102]. Similarly, race alters how depression is linked to obesity [43,44,104,105], self-rated health [6,45,50], chronic medical conditions [14,46,52] and mortality [50,51]. Such race differences may be a consequence of racial differences in the distribution of psychosocial risk and protective factors, or race differences in susceptibilities or vulnerabilities to the effect of risk and protective factors [50,55,56,57]. In the latter view, race may play a major contextual role in shaping vulnerability to the effects of resources such as SES and social support on physical or mental health [18,45,48,104,108,109,110,111,112], in an adaptive response to racism. 

### Limitations

Our study has several limitations. First, we studied depressive symptoms rather than major depressive disorder diagnosed by a clinician, nor did we include the use of anti-depressant medications by the participants as an outcome. Second, we cannot rule out the possibility that psychosocial constructs may not reflect the same aspects across populations [56,108]. Third, we did not control for a number of potential confounders including fatigue, daily activity, cognitive ability and access to the health care system. Fourth, this study exclusively focused on emotional social support and did not include other types of social support. In 1985, House & Kahn differentiated emotional social support from other instrumental and informational support [90]. Instrumental support involves the provision of material aid, whereas informational support refers to the provision of relevant information intended and emotional support involves providing empathy, caring, reassurance and trust [90]. Social support can be seen as flow of psychological and material resources in social network, which is commonly with the intention to benefit members of the social network [95]. We did not control for church attendance because this could be a case of over-adjustment or un-necessary adjustment [113,114]. Future research is needed to explore racial and ethnic differences in the effects of other types of social support on health. Despite the above limitations, using a national sample of older American adults was a strength of this study. Another strength of this study was that we controlled for chronic medical conditions, which may confound the link between social support and depression [5,6,47,48,50,109,115]. The results reported here may also have implications for the reducing racial gaps in treatment of depression in the United States, which has been a challenge for decades [116,117]. 

## 5. Conclusions

To conclude, Black and White older adults differ in their susceptibility to the protective effects of secular and religious social support against depressive symptoms. Differential access and susceptibility to social support may be a mechanism behind the Black—White health paradox in the United States. 

## Figures and Tables

**Table 1:** Descriptive Statistics for the analytic sample, stratified by race and overall.

	Mean	SD	Mean	SD	Mean	SD
	All		Whites		Blacks	
Age	75.14	6.66	75.37	6.82	74.91	6.49
Chronic Medical Conditions (Number)	1.77	1.82	1.74	1.81	1.78	1.83
Depressive symptoms *	1.49	0.69	1.47	0.62	1.52	0.77
Frequency of Church Attendance *	5.73	2.72	5.34	2.91	6.11	2.46
Religious Support *	1.89	0.95	1.73	0.91	2.02	0.96
Secular Support *	0.16	0.62	0.01	0.01	1.92	1.15
	*n*	%	*n*	%	*n*	%
Gender **						
Male	570	38.2	314	41.4	256	34.9
Female	923	61.8	445	58.6	478	65.1
Education (High school diploma)						
Yes	872	59.0	552	73.4	320	44.0
No	607	41.0	200	26.6	407	56.0
Marital Status (Married) **						
No	773	52.2	306	40.5	467	64.3
Yes	708	47.8	449	59.5	259	35.7

* *p* < 0.05, Independent sample t test; ** *p* < 0.05, Chi Square.

**Table 2:** Effects of church attendance on depressive symptoms based on race in older adults using linear regressions.

	*B*(SE)	*p*	*B*(SE)	*p*	*B*(SE)	*p*	*B*(SE)	*p*
	*Model 1* *Pooled Sample Without Interaction*		*Model 2* *Pooled Sample With Interaction*		*Model 3* *Whites*		*Model 4* *Blacks*	
Race (Blacks)	0.00 (0.05)	0.957	0.08 (0.11)	0.308	-	-	-	-
Age	0.05 (0.00)	0.079	0.05 (0.00)	0.084	0.09 (0.00)	0.034	0.02 (0.01)	0.710
Gender (Female)	0.10 (0.05)	0.003	0.1 (0.05)	0.002	0.15 (0.05)	<0.001	0.04 (0.08)	0.368
Education (High school diploma)	−0.07 (0.05)	0.044	−0.07 (0.05)	0.047	−0.04 (0.06)	0.288	−0.07 (0.07)	0.111
Marital Status (Married)	0.01 (0.05)	0.778	0.01 (0.05)	0.787	0.01 (0.06)	0.851	0.00 (0.08)	0.965
Chronic Medical Conditions (Number)	0.23 (0.01)	<0.001	0.23 (0.01)	<0.001	0.26 (0.02)	<0.001	0.20 (0.02)	<0.001
Frequency of Church Attendance	−0.11 (0.01)	<0.001	−0.08 (0.01)	0.031	−0.11 (0.01)	0.012	−0.12 (0.02)	0.010
Frequency of Church Attendance × Race	-	-	−0.09 (0.02)	0.271	-	-	-	-

**Table 3:** Effects of religious social support on depressive symptoms based on race in older adults using linear regressions.

	*B*(SE)	*p*	*B*(SE)	*p*	*B*(SE)	*p*	*B*(SE)	*p*
	*Model 1* *Pooled Sample Without Interaction*		*Model 2* *Pooled Sample With Interaction*		*Model 3* *Whites*		*Model 4* *Blacks*	
Race (Blacks)	0.03 (0.06)	0.496	0.19 (0.11)	0.028	-	-	-	-
Age	0.04 (0.00)	0.272	0.04 (0.00)	0.247	0.09 (0.01)	0.132	0.01 (0.01)	0.824
Gender (Female)	0.10 (0.06)	0.014	0.10 (0.06)	0.011	0.19 (0.07)	0.001	0.04 (0.09)	0.483
Education (High school diploma)	−0.10 (0.06)	0.010	−0.10 (0.06)	0.010	0.06 (0.08)	0.309	−0.02 (0.08)	0.695
Marital Status (Married)	0.02 (0.06)	0.676	0.02 (0.06)	0.723	0.08 (0.04)	0.147	−0.08 (0.04)	0.129
Chronic Medical Conditions (Number)	0.21 (0.02)	<0.001	0.21 (0.02)	<0.001	0.20 (0.02)	<0.001	0.21 (0.02)	<0.001
Religious Social Support	−0.02 (0.03)	0.616	0.07 (0.04)	0.199	−0.10 (0.09)	0.090	−0.10 (0.08)	0.058
Religious Social Support × Race	-	-	−0.21 (0.05)	0.033	-	-	-	-

**Table 4:** Effects of secular social support on depressive symptoms based on race in older adults using linear regressions.

	*B*(SE)	*p*	*B*(SE)	*p*	*B*(SE)	*p*	*B*(SE)	*p*
	*Model 1* *Pooled Sample Without Interaction*		*Model 2* *Pooled Sample With Interaction*		*Model 3* *Whites*		*Model 4* *Blacks*	
Race (Blacks)	0.06 (0.12)	0.353	0.58 (0.23)	<0.001	-	-	-	-
Age	0.05 (0.01)	0.375	0.06 (0.01)	0.308	0.09 (0.01)	0.192	0.04 (0.02)	0.708
Gender (Female)	0.12 (0.10)	0.051	0.14 (0.10)	0.022	0.13 (0.10)	0.092	0.17 (0.27)	0.146
Education (High school diploma)	−0.03 (0.11)	0.659	−0.01 (0.10)	0.812	−0.03 (0.10)	0.689	0.04 (0.28)	0.752
Marital Status (Married)	−0.01 (0.10)	0.852	−0.02 (0.10)	0.763	−0.10 (0.10)	0.167	0.13 (0.27)	0.238
Chronic Medical Conditions (Number)	0.22 (0.03)	<0.001	0.22 (0.03)	<0.001	0.27 (0.03)	<0.001	0.15 (0.08)	0.173
Secular Support	−0.37 (0.05)	<0.001	−0.14 (0.07)	0.051	−0.16 (0.06)	0.033	−0.64 (0.11)	<0.001
Secular Support × Race	-	-	−0.62 (0.10)	<0.001	-	-	-	-
